# Infection par *Helicobacter pylori*: prévalence et facteurs associés dans une population tout venant d’après une recherche par test respiratoire à l’urée marquée au carbone 14

**DOI:** 10.11604/pamj.2021.40.266.22378

**Published:** 2021-12-30

**Authors:** Aboudou Raïmi Kpossou, Homagnissin Benoît Kouwakanou, Carin Ahouada, Rodolph Koffi Vignon, Comlan N’déhougbèa Martin Sokpon, Vincent Zoundjiekpon, Nicolas Kodjoh, Jean Séhonou

**Affiliations:** 1 Clinique Universitaire d’Hépato-Gastro entérologie, Centre National Hospitalier et Universitaire Hubert Koutoukou MAGA (CNHU-HKM), Cotonou, Bénin,; 2 Service d´Hépato-Gastroentérologie, CHU Mohammed VI de Marrakech, Marrakech, Maroc,; 3 Service de médecine, Hôpital de Zone d´Allada, Allada, Bénin,; 4 Service d´Hépato-Gastroentérologie et Gériatrie, Hôpital Universitaire d´Olomouc, Olomouc, République Tchèque,; 5 Clinique les Archanges, Agblangandan - Sèmè Podji, Cotonou, Bénin

**Keywords:** *Helicobacter pylori*, prévalence, test respiratoire, facteurs associés, *Helicobacter pylori*, prevalence, respiratory test, associated factors

## Abstract

**Introduction:**

l´infection à Helicobacter pylori (H. pylori) est fréquente dans les pays en voie de développement comme le Bénin. Ce germe peut favoriser la survenue d´affections gastroduodénales, allant de la gastrite au cancer gastrique. Les différentes études réalisées au Bénin sur cette bactérie ont utilisé des méthodes telles que la sérologie, l´étude anatomo-pathologique de biopsies ou la recherche d´antigène dans les selles. La présente étude avait pour but d´évaluer la prévalence d´infection par H. pylori et les facteurs associés à cette infection en utilisant un test respiratoire.

**Méthodes:**

il s´agissait d´une étude prospective descriptive sur 150 patients qui ont réalisé le test respiratoire à l´urée marquée au carbone 14. Seul les patients admis pour test respiratoire et ayant donné leur consentement étaient inclus. Une fiche d´enquête a été remplie au fur et à mesure. Une analyse univariée par régression logistique simple a permis d´identifier les facteurs associés à l´infection par H. pylori à un seuil de 0,05. La stratégie d´analyse multivariée a consisté à inclure dans le model toutes les variables dont la valeur de p est inférieure à 0,20. La procédure manuelle descendante a été utilisée jusqu´à obtenir le modèle final qui a permis de retenir de facteurs associés avec des rapports de cotes ajustés.

**Résultats:**

l´âge moyen était de 44,4±15,8 ans; avec des extrêmes de 5 ans et 84 ans. Les hommes représentaient 54% de la population d´étude. Des 150 sujets, 82 (57,8%) avaient un niveau d´instruction supérieur contre 8 (5,6%) non scolarisés, 116 (80,6 %) vivaient en couple, 24 (36%) vivaient dans une chambre de plus de 10 personnes et 84 (59,6%) étaient des chrétiens. Les principaux motifs ayant conduit à la réalisation de test respiratoire étaient: douleurs abdominales mal systématisées (53,3%; 70/150), douleurs épigastriques provoquées (35,3%; 53/150), les épigastralgies (20,7%; 31/150), un syndrome ulcéreux (16%; 24/150). La prévalence d´H. pylori par test respiratoire au sein de la population étudiée était de 34,7% (52/150). En analyse multivariée, les variables significativement associées à l´infection à l´H. pylori étaient: l´âge moyen [AOR (95%IC) = 1,02; OR (95% IC) = 1,00-1,05 et p = 0,01] et la notion d´un traitement antérieur d´éradication de l´H. pylori[AOR (95%IC) = 4,79; OR (95% IC) = 1,50-13,86 et p = 0,006]. Aucune comorbidité étudiée n´était associée à l´infection par H. pylori dans notre série.

**Conclusion:**

la prévalence d´H. pylori trouvée par cette méthode est faible (34,7%). Elle est significativement associée à l´âge moyen et à la notion d´un traitement antérieur d´éradication de l´H. pylori.

## Abstract (en)

**Introduction:**

Helicobacter pylori (H. pylori) infection is common in developing countries such as Benin. This germ may cause the development of gastroduodenal diseases, ranging from gastritis to gastric cancer. Several studies on this bacterium carried out in Benin have used serology, anatomopathological examination of biopsies or antigen detection test on stool sample collections. The purpose of this study was to assess the prevalence of H. pylori infection and factors associated with H. pylori infection using a respiratory test. The bacterium could cause chronic inflammation of the gastric mucosa and peptic ulcer disease or, more rarely, gastric cancer or lymphoma.

**Methods:**

we conducted a prospective, descriptive study of 150 patients who performed carbon-14 urea breath test. Only patients admitted for respiratory test and who gave their consent were included. A survey was completed by all patients during the study. Simple univariate logistic regression analysis identified factors associated with H. pylori infection at a threshold of 0.05. Multivariate analysis included in the model all variables with a p-value less than 0.20. Manual top-down procedure was used until the final model was obtained, which allowed the retention of adjusted odds ratios for factors associated.

**Results:**

the average age of patients was 44.4±15.8 years, ranging from 5 to 84 years; men accounted for 54% of the study population. Of the 150 subjects, 82 (57.8%) had higher education level, compared with 8 (5.6%) who were not educated, 116 (80.6%) lived with a partner, 24 (36%) lived in a room with more than 10 people and 84 (59.6%) were christians. The main reasons why respiratory tests were performed were: poorly systematized abdominal pain (53.3%; 70/150), epigastric induced pain (35.3%; 53/150), epigastralgia (20.7%; 31/150), ulcer syndrome (16%; 24/150). Respiratory test showed that the prevalence of H. pylori in the study population was 34.7% (52/150). In multivariate analysis, the variables significantly associated with H. pylori infection were: mean age [aOR (95% CI) = 1.02; OR (95% CI) = 1.00-1.05 and p = 0.01] and prior treatment for H. pylori eradication [aOR (95% CI) = 4.79; OR (95% CI) = 1.50-13.86 and p = 0.006]. No comorbidity was associated with H. pylori infection in our series.

**Conclusion:**

the prevalence of H. pylori using this method was low (34.7%). It was significantly associated with mean age and prior treatment for H. pylori eradication.

## Introduction

L´infection par *H. pylori* est très répandue avec près de 50 % de la population mondiale touché [1,2]. Le taux d´infection varie en fonction de nombreux critères comme l´âge, l´origine géographique et les conditions de vie [2-5]. Dans les pays en voie de développement, on estime que 80% de la population y est touchée avant l´âge de 20 ans [6]. Certaines études réalisées en Afrique de l´Ouest montrent que sa prévalence moyenne est supérieure à 70% [7-9]. En revanche, dans les pays développés, un adulte sur 5 est infecté à l´âge de 20 ans [2].

*H. pylori* est une bactérie gram négatif, uréase positive qui ne vit que dans l´estomac humain [10,11]. L´homme peut être contaminé par deux moyens: la voie orale ou par les selles. Le mode de transmission implique la proximité, c´est pourquoi *H. pylori* se transmet le plus souvent au sein d´une même famille, en particulier dans le sens parent-enfant ou entre enfants [5]. Parmi les facteurs favorisant sa transmission, on retrouve la vie en collectivité, le partage des couverts, ou l´habitude de mastiquer les aliments donnés aux nourrissons [4]. Les facteurs liés aux mauvaises conditions socio-économiques et à l´hygiène sont également déterminants: absence de WC dans le logement, absence d´eau courante, mauvaise hygiène des mains après avoir été aux toilettes [4].

Elle constitue la principale cause des gastrites chroniques, des ulcères gastroduodénaux (UGD), des syndromes dyspeptiques et de deux types de néoplasies gastriques: l´adénocarcinome et le lymphome du MALT (Mucosa Associated Lymphoid Tissue) [12]. De nombreuses méthodes ont été validées pour son diagnostic: les tests invasifs avec biopsies gastriques lors de la gastroscopie (histologie, culture, test rapide à l´uréase), soit par des tests non invasifs (test respiratoire, détection antigénique dans les selles, sérologie) [13-15]. Le test respiratoire reste le meilleur test diagnostique non invasif de l´infection, tandis que la sérologie est le seul test non affecté par la modification des conditions locales gastriques [12].

Au Bénin, aucune étude épidémiologique ne s´est encore basée sur le test respiratoire pour déterminer la prévalence de l´infection à l´*H. pylori*. Le but de cette étude est de déterminer la prévalence et les facteurs associés à l´infection à *H. pylori* chez des patients admis dans une clinique privée pour un test respiratoire à l´urée marquée à visée de diagnostic et/ou de contrôle de l´éradication.

## Méthodes

**Conception et contexte de l´étude:** il s´agit d´une étude transversale descriptive avec un recueil prospectif des données. Elle a été réalisée sur une période de 12 mois, allant de juillet 2017 à juin 2018. Cette étude a eu pour cadre la Clinique les Archanges, une clinique privée située dans la commune de Sèmè Podji, (à la périphérie Sud de Cotonou) au Bénin.

**Population d´étude:** elle est constituée de tous les patients reçus dans la période d´étude pour la réalisation d´un test respiratoire à l´urée marquée dans le but du diagnostic ou du contrôle après éradication d´une infection à *H. pylori*. Le consentement éclairé verbal des patients était obtenu avant l´inclusion. Seuls les patients ayant accepté de participer volontairement à l´enquête étaient inclus dans cette étude.

**Collecte des données:** il s´agissait d´une collecte prospective qui s´est déroulée pendant la période d´étude. Une fiche d´enquête avait été utilisée pour le recueil des données. La variable dépendante était le résultat du test respiratoire à l´urée marquée au carbone 14 (14C). Les variables indépendantes étaient sociodémographiques, cliniques, endoscopiques et thérapeutiques. Après le remplissage du questionnaire, nous procédions au test respiratoire. Des conditions à respecter pour l´examen étaient: le patient devrait être à jeun, ou à défaut attendre deux heures (2h) après le repas si le patient avait déjà mangé; il devrait avoir arrêté tout traitement antibiotique depuis au moins 4 semaines et tout inhibiteur de la pompe à protons ou autre anti-sécrétoire depuis au moins 2 semaines. Le résultat était consigné sur la fiche de collecte.

**Définitions des variables:** la variable dépendante était représentée par le résultat du test respiratoire tandis que les variables indépendantes étaient représentées par les données socio démographiques (l´âge, le sexe, la profession, le niveau d´instruction, le statut matrimonial, le nombre de personnes vivant sous un même toit), les antécédents et comorbidités, les données cliniques, endoscopiques et thérapeutiques en rapport avec une infection à *H. pylori*.

**Méthodes et analyse statistique:** les données ont été saisies dans Epidata. Les variables catégorielles ont été exprimées en pourcentages. Les variables continues ont été exprimées en moyenne et son écart type. Une analyse univariée par régression logistique simple a permis d´identifier les facteurs associés à l´infection de l´*H. pylori* à un seuil de 0,05. La stratégie d´analyse multivariée a consisté à inclure dans le model toutes les variables dont la valeur p est inférieure à 0,20. La procédure manuelle descendante est utilisée jusqu´à obtenir le modèle final qui a permis de retenir de facteurs associés avec des rapports de cotes ajustés. L´analyse statistique est faite dans SAS studio.

## Résultats

### Caractéristiques de la population étudiée

Notre étude a porté sur 150 patients; l´âge moyen était de 44,4±15,8 ans, avec des extrêmes de 5 ans et 84 ans. Les hommes représentaient 54% (81 hommes) de la population d´étude. Par ailleurs, 57,8% (82 patients) de la population d´étude avaient un niveau d´instruction supérieur contre 5,6% (8 patients) qui étaient non scolarisés, 80,6 % (116 patients) vivaient en couple, 36% (24 patients) vivent dans une chambre de plus de 10 personnes et 59,6% (84 patients) étaient des chrétiens. Comme comorbidités, l´hypertension artérielle (HTA) était retrouvée chez 30,7% (31 patients) tandis que 17,2% (23 patients) de la population étudiée rapportaient un antécédent familial au 1er degré d´ulcère ou d´épigastralgie (Tableau 1, Tableau 1 (suite)). Les principales indications du test respiratoire étaient des douleurs abdominales mal systématisées (53,3% soit 70 patients), une sensibilité ou douleur provoquée épigastrique (35,3% soit 53 patients), des épigastralgies (20,7% soit 31 patients), des syndromes ulcéreux (16% soit 24 patients), un amaigrissement (14,7% soit 22 patients) et une dyspepsie douloureuse (6,7% soit 10 patients).

**Tableau 1:** caractéristiques sociodémographiques, cliniques et endoscopiques de l´infection à *Helicobacter pylori*

Caractéristiques	n		%
Age			
Age moyen ET	44,4±15,8 ans		
Sexe			
Homme	81		54,0
Femme	69		46,0
Niveau d´étude *			
Primaire	10		7,0
Secondaire	42		29,6
Supérieur	82		57,8
Non scolarisé	5		3,5
Autres	3		2,1
Statut matrimonial			
Célibataire	24		16,7
En couple	116		80,6
Divorcé(e)	0		0
Autres	4		2,8
Religion			
Islam	18		12,8
Christianisme	84		59,6
Animisme	6		4,3
Aucune	33		23,4
Nombre de personnes vivant sous le même toit			
≤ 4	77		51,3
5-10	37		24,7
≥ 10	36		24,0
Antécédents et comorbidités *			
Obésité			
Oui	13		8,7
Non	137		91,3
Diabète			
Oui	10		6,9
Non	130		89,0
Non recherché	6		4,1
HTA			
Oui	31		30,7
Non	59		58,4
Non recherché	11		10,9
Cirrhose			
Oui	2		98,7
Non	148		1,3
VIH			
Oui	2		1,4
Non	133		92,4
Non recherché	9		6,3
Tabac			
Oui	4		2,7
Non	133		90,5
Non recherché	10		6,8
Alcool			
Oui	5		3,4
Non	137		93,8
Non recherché	4		2,7
ATCD familial au 1^er^ degré d´ulcére ou d´épigastralgies			
Oui	23		17,2
Non	80		59,7
Non recherché	31		23,1
ATCD familial au 1^er^ degré de cancer gastrique			
Oui	10		7,5
Non	83		62,4
Non recherché	40		30,1
**Données cliniques**			
Dyspepsie douloureuse			
Oui		10	6,7
Non		140	93,3
Epigastralgies			
Oui		31	20,7
Non		119	79,3
Dyspepsie non douloureuse			
Oui		3	2,0
Non		147	98,0
Syndrome ulcéreux			
Oui		24	16,0
Non		126	84,0
Douleurs abdominales mal systématisées			
Oui		70	53,3
Non		80	46,7
Hématémèse ou mélaena			
Oui		1	0,7
Non		149	99,3
Amaigrissement			
Oui		22	14,7
Non		128	85,3
Sensibilité ou douleur provoquée épigastrique			
Oui		53	35,3
Non		97	64,7
**Test respiratoire**			
Indications de réalisation du test respiratoire			
Diagnostic initial,		131	87,3
Contrôle d´éradication		19	12,7
**Principales données endoscopiques**			
Endoscopie déjà faite une fois			
Oui		104	69,3
Non		46	30,7
Gastrite uniquement antrale			
Oui		12	8,0
Non		138	92,0
Gastrite uniquement fundique			
Oui		4	2,7
Non		146	97,3
Pangastrite			
Oui		8	5,3
Non		142	94,7
Gastrite nodulaire			
Oui		3	2,0
Non		147	98,0
Ulcère gastrique			
Oui		5	3,3
Non		145	96,7
Ulcère duodénal			
Oui		11	7,3
Non		139	92,7
Tumeur gastrique			
Oui		3	2,0
Non		147	98,0
**Données thérapeutiques**			
Traitement antérieur			
Oui		31	20,7
Non		119	79,3
Traitement fait pour Hélicobacter pylori			
Oui		2	1,3
Non		148	98,7

(*): le total ne fait pas 150 du fait des données manquantes

### Prévalence de l´infection par *H. pylori* et facteurs associés

La prévalence d´*H. pylori* par test respiratoire au sein de la population étudiée était de 34,6% (52 patients testés positif à *H. pylori* sur 150). En analyse univariée, les variables suivants: âge, la situation matrimoniale (en couple), plus de 10 personnes vivants sous un même toit, absence d´antécédent familial d´ulcère ou d´épigastralgie au 1er degré, la présence d´une gastrite antrale en l´endoscopie digestive haute et une notion de traitement antérieur d´éradication d´*H. pylori*étaient significativement associés à l´infection à *H. pylori*. En analyse multivariée, les variables qui étaient significativement associées à l´infection à l´*H. pylori* étaient: l´âge moyen [AOR (95%IC) = 1,02; OR (95% IC) = 1,00-1,05 et p = 0,01] et la notion d´un traitement antérieur d´éradication de l´*H. pylori* [AOR (95%IC) = 4,79; OR (95% IC) = 1,>50-13,86 et p = 0,006] (Tableau 2, Tableau 2 (suite))

**Tableau 2:** prévalence et facteurs associés à l´infection à *Helicobacter pylori*

	H. pylori		OR(95%IC)	p	AOR(95%IC)	p
Oui n(%)	Non n(%)
Caractéristiques*						
Age						
Age moyen±ET	40,2(17,4)	46,6(14,5)	1,02(1,00-1,05)	0,01	1,02(1,00-1,05)	0,01
Sexe						
Homme	25(30,9)	56(69,1)	0,69(0,35-1,36)	0,28		
Femme	27(39,1)	42(60,9)	1			
Niveau d´étude*						
Primaire	2(20,0)	8(80,0)	1			
Secondaire	18(42,9)	24(57,14)	0,33(0,06-1,76)	0,19		
Supérieur	28(34,2)	54(65,85)	0,48(0,09-2,42)	0,37		
Non scolarisé	1(20,0)	4(80)	1(0,06-14,64)	1		
Autres	0	3(100)	-	-		
Statut matrimonial						
Célibataire	14(58,3)	10(41,7)	1			
En couple	33(28,5)	83(71,6)	3,52(1,42-8,71)	0,006		
Autres	2(50,0)	2(50,0)	1,40(0,16-11,67)	0,75		
Réligion						
Islam	7(38,9)	11(61,1)	1			
Christianisme	30(35,1)	54(64,3)	1,14(0,40-3,26)	0,79		
Aucune	0	6(100)	-	-		
Autres	12(36,4)	21(63,6)	1,11(0,34-3,63)	0,85		
Nombre de personnes vivant sous le même toit						
≤ 4	30(39,0)	47(61,0)	1			
5-10	16(43,2)	21(56,8)	0,83(0,37-1,85)	0,66		
≥ 10	6(16,7)	30(83,3)	3,19(1,18-8,58)	0,02		
Antécédents et comorbidités*						
Obésité						
Oui	2(15,4)	11(84,6)	3,16(0,67-14,83)	0,14		
Non	50(36,5)	87(63,5)	1			
Diabète						
Oui	4(40,0)	6(60,0)	1			
Non	45(34,6)	85(65,4)	1,25(0,33-4,69)	0,73		
Non recherché	2(33,3)	4(66,7)	1,33(0,16-11,07)	0,79		
HTA						
Oui	7(22,6)	24(77,4)	1			
Non	24(40,7)	35(59,3)	0,42(0,15-1,14)	0,09		
Non recherché	2(18,2)	9(81,8)	1,31(0,22-7,54)	0,76		
Cirrhose						
Oui	0	2(100)	-	-		
Non	52(35,1)	96(64,9)	1			
VIH						
Oui	1(50,0)	1(50,0)	1			
Non	45(33,8)	88(66,2)	1,95(0,12-31,99)	0,63		
Non recherché	5(55,6)	4(44,4)	0,80(0,03-17,19)	0,88		
Tabac						
Oui	1(25,0)	3(75,0)	1			
Non	47(35,3)	86(64,7)	0,61(0,06-6,03)	0,67		
Non recherché	4(40,0)	6(60,0)	0,5(0,03-6,68)	0,60		
Alcool						
Oui	1(20,0)	4(80,0)	1			
Non	49(35,8)	88(64,2)	0,44(0,04-4,13)	0,47		
Non recherché	1(25,0)	3(75,0)	0,75(0,03-17,50)	0,85		
ATCD familial au 1er degré d´ulcère ou d´épigastralgies						
Oui	4(17,4)	19(82,6)	1			
Non	32(40,0)	48(60,0)	0,31(0,09-1,01)	0,05		
Non recherché	10(32,3)	21(67,7)	0,44(0,11-1,64)	0,22		
ATCD familial au 1er degré de cancer gastrique						
Oui	5(50,0)	5(50,0)	1			
Non	32(38,6)	51(61,5)	1,59(0,42-5,94)	0,48		
Non recherché	12(30,0)	28(70,0)	2,33(0,56-9,57)	0,23		
**Données cliniques**						
Dyspepsie douloureuse						
Oui	3(30,0)	7(70,0)	1,26(0,31-5,07)	0,74		
Non	49(35,0)	91(65,0)	1			
Epigastralgies						
Oui	7(22,6)	24(77,4)	2,08(0,83-5,23)	0,11		
Non	45(37,8)	74(62,2)	1			
Dyspepsie non douloureuse						
Oui	0	3(100)	-	-		
Non	52(35,4)	95(64,6)	1			
Syndrome ulcéreux						
Oui	5(20,8)	19(79,2)	2,26(0,79-6,45)	0,12		
Non	47(37,3)	79(62,7)	1			
Douleurs abdominales mal systématisées						
Oui	29(41,4)	41(58,6)	0,57(0,28-1,12)	0,10		
Non	23(28,8)	57(71,3)	1			
Hématémèse ou mélaena						
Oui	1(100)	0	-	-		
Non	51(34,2)	98(65,8)	1			
Amaigrissement						
Oui	9(40,9)	13(59,1)	0,73(0,29-1,84)	0,50		
Non	43(33,6)	85(66,4)	1			
Sensibilité ou douleur provoquée épigastrique						
Oui	14(26,4)	39(73,6)	1,79(0,86-3,73)	0,11		
Non	38(39,2)	59(60,8)	1			
**Principales données endoscopiques**						
Endoscopie déjà faite une fois						
Oui	36(34,6)	68(65,4)	1,00(0,48-2,08)	0,98		
Non	16(34,8)	30(65,2)	1			
Gastrite uniquement antrale						
Oui	1(8,3)	11(91,7)	6,44(0,80-51,41)	0,07		
Non	51(37,0)	87(63,0)	1			
Gastrite uniquement fundique						
Oui	2(50,0)	2(50,0)	0,52(0,07-3,80)	0,52		
Non	50(34,3)	96(65,8)	1			
Pangastrite						
Oui	5(62,5)	3(37,5)	0,29(0,06-1,29)	0,10		
Non	47(33,1)	95(66,9)	1			
Gastrite nodulaire						
Oui	0	3(100)	-	-		
Non	52(35,4)	95(64,6)	1			
Ulcère gastrique						
Oui	2(40,0)	3(60,0)	0,78(0,12-4,88)	0,79		
Non	50(34,5)	95(65,5)	1			
Ulcère duodénal						
Oui	2(18,2)	9(81,8)	2,52(0,52-12,16)	0,24		
Non	50(36,0)	89(64,0)	1			
Tumeur gastrique						
Oui	1(33,3)	2(66,7)	1,06(0,09-11,99)	0,96		
Non	51(34,7)	96(65,3)	1			
**Données thérapeutiques**						
Traitement antérieur						
Oui	4(12,9)	27(87,1)	4,56(1,50-13,86)	0,007	4,79(1,56-14,72)	0,006
Non	48(40,3)	71(59,7)	1		1	
Traitement fait pour H. pylori						
Oui	1(50,0)	1(50,0)	0,52(0,03-8,58)	0,65		
Non	51(34,5)	97(65,5)	1			

(*): le total ne fait pas 150 du fait des données manquantes

## Discussion

Notre étude avait pour objectif principal de déterminer la prévalence et les facteurs associés à l´infection à *H. pylori* à l´aide d´un test respiratoire à l´urée marquée au carbone 14. La prévalence d´*H. pylori* au sein de la population étudiée était de 34,7%. Cette prévalence est très basse par rapport à celle rapportée dans les études antérieures, notamment au Bénin respectivement en 2005 [16] où la prévalence était de 75,4% en zone péri-urbaine et de 72,2% en zone rurale et de 71,1% en 2015 [7]; au Nigéria [8] où elle était de 80%; au Cameroun en 2012 [9] où elle était de 72,5% et au Maroc en 2010 [17] où elle était de 69% bien que les méthodes utilisées ne soient pas les mêmes. Notre résultat est similaire à ceux d´Itoudi *et al*. au Gabon en 2013 [18] qui rapportaient une prévalence de 36% et déclaraient le Gabon comme un pays Africain à faible prévalence d´*H. pylori*. A en croire Kalach *et al*. [19], cette baisse de la prévalence d´*H. pylori* est un constat général fait dans le monde, tant chez l´adulte que chez l´enfant. Notons toutefois que cette faible prévalence dans notre travail peut être liée à une sensibilité faible du test respiratoire utilisé.

Parlant des méthodes, plusieurs moyens sont utilisés pour le diagnostic de l´infection à l´*H. pylori*. Les méthodes invasives nécessitant une biopsie gastrique après une endoscopie digestive haute (anatomie pathologique, test à l´uréase, la culture et l´amplification génique) [12]. Les techniques non invasives sont principalement utilisées quand la gastroscopie n´est pas indispensable, ou en complément des méthodes sur biopsies et au nombre desquelles on peut citer la sérologie *H. pylori*, le test respiratoire à l´urée marquée et la détection antigénique d´*H. pylori* dans les selles. La sérologie utilise la méthode Elisa (Enzyme linked immuno-sorbent assay). La sérologie détecte une infection active mais aussi une infection guérie et dont persiste encore les titres d´anticorps. Elle ne peut être utilisée pour le suivi après traitement. La sérologie n´a aucune place dans la pratique quotidienne et est donc réservée aux études épidémiologiques [12,20]. Le test respiratoire à l´urée est le plus efficace pour contrôler l´éradication d´*H. pylori* [13,15]. Également performant pour le diagnostic avant traitement, chez l´adulte comme chez l´enfant [13], il détecte une infection active par la mise en évidence de l´activité uréasique d´*H. pylori*. Il peut être remplacé par la détection antigénique dans les selles, de performances équivalentes en utilisant un Elisa à base d´anticorps monoclonaux [13]. Ces 2 deux tests ont une sensibilité diminuée si hémorragie digestive, estomac opéré. Le choix de test respiratoire pour étudier la prévalence de *H. pylori* se justifie bien dans la mesure où cette technique nous permet d´avoir une prévalence des infections actives mais aussi le taux de guérison pour l´éradication de l´*H. pylori* après traitement.

Les facteurs associés à l´infection à l´*H. pylori* dans notre étude sont représentés par l´âge moyen et la notion d´un traitement antérieur d´éradication de l´*H. pylori*. Par rapport à l´âge, la moyenne d´âge de prévalence élevée est la quarantaine et semble montrer une similitude entre les résultats rapportés par d´autres études antérieures [7,9,21]. D´autres études ont rapporté que la prévalence augmentait avec l´âge dans les pays développés alors qu´elle est acquise dans l´enfance dans les pays en voie de développement (avec plus de 80% de population contaminés avant l´âge de 20ans) [6]. Autre facteur associé à l´infection dans notre étude est la notion d´un traitement antérieur d´éradication de l´*H. pylori* et pourrait s´expliquer par la résistance de certaines souches à la quadrithérapie concomitante comme le rapportent Gressot P *et al*. en 2019 [22].

D´autres facteurs associés ont été rapportés dans la littérature comme la promiscuité, le nombre de personnes dormant sous un même toit, la taille de la fratrie, un manque d’hygiène, le sexe et le niveau d´instruction [6,21,23]. Ces différents facteurs n´ont pas été retrouvés associés de façon significative à l´infection à *H. pylori* dans notre série. La promiscuité, la proximité et une hygiène plus ou moins défectueuse constituent des conditions favorables à la transmission de *H. pylori* de personne à personne [3-5]. Le mode de transmission de l´*H. pylori* implique la proximité, c´est ce qui explique sa propagation le plus souvent au sein d´une même famille, en particulier dans le sens parent-enfant ou entre enfants car ces derniers sont plus sensibles [5]. Il faut aussi le rappeler que le taux élevé d´infection est inversement proportionnel au niveau d´instruction des mères [21]. Aucune différence significative n´est observée par rapport au sexe, même constat fait dans certaines études [7,9,18,24]. Cependant, certains auteurs avaient rapporté une prédominance masculine [23].

Notre étude a l´intérêt d´être la première à évaluer la prévalence d´*H. pylori* au Bénin en utilisant le test respiratoire. Elle présente toutefois des limites, notamment des données manquantes par rapport aux antécédents des patients, aux résultats endoscopiques et la précision des traitements antérieurs d´éradication.

## Conclusion

L´infection à *H. pylori* demeure endémique dans les pays en voie de développement. La prévalence d´*H. pylori*. trouvée par le test respiratoire dans notre travail est faible. Elle est significativement associée à l´âge moyen et la notion d´un traitement antérieur d´éradication de l´*H. pylori*. Il est souhaitable qu´une autre étude utilisant le test respiratoire soit encore éditée avec une augmentation si possible de la taille de l´échantillon. Une étude comparant le test respiratoire utilisé à la recherche d´*H. pylori* par l´examen anatomopathologique serait intéressante pour conforter ces données.

### Etat des connaissances sur le sujet

- *L´infection par Helicobacter pylori est fréquente en Afrique subsaharienne, y compris au Bénin (71,1% d´après une étude antérieure par examen anatomo-pathologique de biopsies gastrique);*
- *La contamination se fait généralement dans la petite enfance, notamment à la faveur de la promiscuité*.

### Contribution de notre étude à la connaissance

- *La prévalence de l´infection par H. pylori d´après une recherche par test respiratoire à l´urée marquée chez des patients admis pour diagnostic ou contrôle d´éradication est faible: 34,7%;*
- *Les facteurs significativement associés à cette infection sont: l´âge moyen et la notion d´un traitement éradicateur antérieur*.
